# Neurological update: consult the neurosurgical oracle for a standard operating procedure

**DOI:** 10.1007/s00415-022-11090-2

**Published:** 2022-04-05

**Authors:** Taco Goedemans, Janneke D. M. Verberk, Pepijn van den Munckhof, Dennis R. Buis, W. Peter Vandertop, Antonius M. de Korte

**Affiliations:** 1 Department of Neurosurgery, Amsterdam UMC location University of Amsterdam, Meibergdreef 9, Amsterdam, The Netherlands; 2 Department of Medical Microbiology and Infection Prevention, University Medical Center Utrecht, Utrecht University, Heidelberglaan 100, Utrecht, The Netherlands

**Keywords:** Consensus, Delivery of health care, Delphi method, Neurosurgical procedures, Standard operating procedures, Surveys and questionnaires

## Abstract

Standard operating procedures (SOPs) contain general instructions and principles to standardize care, to improve effective and safe healthcare. Developing new, or updating current, SOPs is, however, challenging in fields where high-level evidence is limited. Still, SOPs alone have been shown to result in less complications. In this narrative review, we describe the process of creating a consensus-based SOP that is pragmatic for clinical practice since it can be created regardless of the current level of evidence. Through live audience engagement platforms, a group of experts will be able to both anonymously respond to a created questionnaire, and (subsequently) discuss the results within the same meeting. This modified Digital Delphi method as described here can be used as a tool toward consensus-based healthcare.

## Introduction

In 2020, we presented a 13-year single-center observational cohort study including patients undergoing cranioplasty after decompressive craniectomy [1]. Similar to many published studies of comparably sized cohorts, post-operative complications occurred in 23% of patients, which makes this high rate of complications a serious point of attention [1–7].

As with many surgeries, the surgical method of cranioplasty is not standardized and surgeons perform their procedures in different ways. This makes it difficult to assess whether the way in which the surgery is performed affects the complication rate. Performing the surgery in a standardized way can help identify what parts of surgery improve or worsen the outcome and can serve as a benchmark for further changes. Uniform, standardized working procedures alone have been shown to result in less complications [8–10]. Creating uniformity in surgical method can be done by implementing a Standard operating procedure (SOP), in which specific parts of a surgical procedure are standardized.

Reaching consensus on surgical methods can however prove to be very difficult, especially if the level of evidence about different (peri)operative strategies is limited. In this Update section, we describe how consensus may be reached within a group of experts/neurosurgeons and how to create (or update) a SOP, inspired by the Delphi method.

## Delphi method

Neurosurgery is a medical specialty with ancient roots. For example, the medical application of trephination started in the Greek era where the neurosurgical oracle called Hippocrates described the procedure in detail [11]. Like many (neuro)surgical procedures, a rich history of passing on knowledge from oracle to student forms the base of nowadays common practice (expert opinion). Randomized controlled trials are often lacking, i.e. due to a low volume of cases within the neurosurgical field, making it difficult to reach adequate statistical power. With the passage of time more oracles arise, sometimes even spreading their different prophecies within the same institution. Considering this variety, how does one reach consensus and create uniformity?

To address this issue, the answer is, again, to be found in ancient Greece. In the 1950s, Norman Dalkey and Olaf Helmer developed a method to find consensus within a group of experts on health issues and topics where minimal information or agreement prevails [12, 13]. They called it the Delphi method, named after the ancient Greek *Oracle of Delphi*: using (typically) three rounds of questionnaires, with a moderator collecting and processing the output of multiple experts, until final consensus is reached within the group. Anonymity in combination with the ability of experts to respond to and revise their answer in view of the previous responses from other panel members, makes this method a successful tool to create consensus [14, 15]. However, in its original form, the method is a time-consuming procedure, lowering the adherence to questionnaire rounds and thus the chance on successful outcomes useful in healthcare. Fortunately, new technological tools can help overcome these problems.

## Live audience engagement platforms: a modified Digital Delphi method

Through ‘live audience engagement platforms’, an online meeting can be arranged in which the moderator presents a questionnaire to a group of experts. Each expert registers his/her presence at the start of the meeting using a smartphone or computer, after which they can anonymously fill in their answers and comments to the questionnaire. Once everyone has responded, the group results can directly be presented, for example in graphical form, and discussed with the group during the online session. This process can be repeated until consensus is reached. This platform type optimizes time management, while the anonymous and interactive characteristics of the Delphi Method are maintained. Examples of platforms are AhaSlides (AhaSlides PTE. LTD., Singapore), Glisser (Glisser LTD, London, United Kingdom), and Menti (Mentimeter, Stockholm, Sweden).

In the next subheadings, we give an overall, step-by-step explanation how to apply this modified Digital Delphi method, from collecting information on surgical methods to the actual implementation of the consent-based protocol. Table 1 shows the key phases and responsible persons for each step within this method. A real-life example is presented in the box below.

**Table 1:** Flow-diagram showing key phases involved in modified Digital Delphi method to create a consensus-based Standard Operating Procedure (SOP)

Creating a consensus-based SOP		
N	Personnel	Point of action
*Phase 1: preparatory steps*		
1.1	Staff member	State scope and extent of SOP, Appoint moderator
1.2	Moderator	Create overview of procedure: review expert’s practice and literature
1.3	Moderator	Define criteria for participation and select and invite group of experts meeting these criteria
1.4	Moderator	Develop questionnaire, send to group of experts
	Group of experts	Review questionnaire, form opinion, revise questions if needed
*Phase 2: interactive session*		
2.1	Moderator	Define level of consensus, e.g., ≥ 80%
2.2	Moderator	Lead interactive session, record answers and discussions
	Group of experts	Answer question anonymously first, motivate answers anonymously second, repeat question again third, discuss results fourth
*Phase 3: consensus based SOP*		
3.1	Moderator	Create SOP based on expert’s input during phase 2, send draft to group of experts, and organize interactive session to discuss SOP
	Group of experts	Review SOP draft, attend interactive session to discuss and/or pledge adherence to SOP
	Moderator	Implement SOP for clinical routine (if agreement exists)
3.2	Moderator	Evaluate SOP effectiveness: record patient outcomes
	Group of experts	Review executability of SOP, address any issues to moderator
*Follow-up*		
	Moderator	Start next periodic SOP revision at point 1.2 after adequate follow-up (e.g., one-yearly)

## Phase 1: preparatory steps

### 1.1 State scope of SOP, appointment of moderator

First, the scope and extent of the SOP should be stated beforehand. Second, a moderator should be appointed. This person does not have to be an expert on the topic but preferably someone who has some experience with the surgical procedure. For example, this can be a resident in training, since a resident typically experiences and observes the variety in surgical methods between attending (neuro)surgeons.

### 1.2 Problem identification: determine current surgical methods, evaluate literature

The moderator creates an overview of the steps of the surgical procedure by assessing (the differences in) practice among the experts, and current literature. In case an SOP already exists, the SOP should be evaluated by the moderator and compared routinely with the newest insights as described in literature, for example every year.

### 1.3 Select group of experts

It is important to clearly define characteristics of those who will be part of the expert group. Since a heterogenic group is more likely to cover all aspects and opinions related to a certain topic, it is advised to include all stakeholders/experts who have knowledge and affinity with the topic. Afterward, or during the session, subgroup analysis can be performed, based on certain characteristics, such as the years of (surgical) expertise or function (i.e., type of physician, (scrub)nurse).

### 1.4 Develop questionnaire

**Fig. 1:**
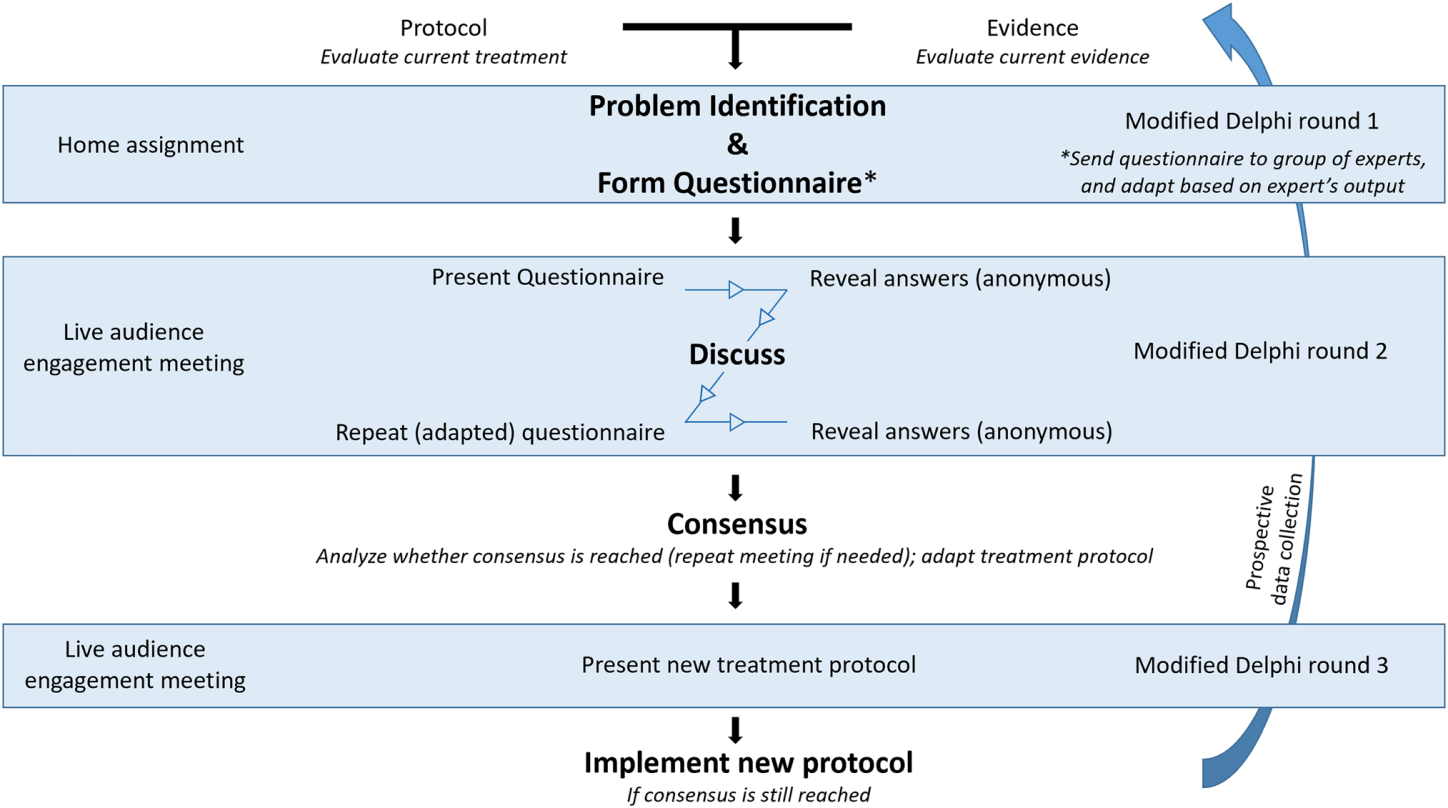
Flow chart describing a modified Digital Delphi method to generate and implement a consensus-based SOP

Subsequently, based on the previous steps, a questionnaire is developed by the moderator. In this questionnaire, points of attention resulting from evaluation of current practice, the current protocol (if applicable) and literature are grouped in overarching themes, and translated into questions to investigate how to deal with them. This questionnaire and literature (if applicable) should be sent to the group of experts before starting the consensus meeting, so the experts will have time to form their opinion on the subject. Furthermore, they will be able to revise the questions or answer possibilities (if needed) to make them clinically more relevant. (Fig. 1, modified Digital Delphi round 1).

## Phase 2: answer questionnaire and discuss topic during an interactive session

### 2.1 Defining consensus

After the questionnaire is sent to the group of experts, the next phase is to try to reach consensus by collecting and discussing the experts’ opinions during an interactive session. Before take-off, consensus criteria should be defined, for example an agreement of ≥ 80% within the group of experts [16, 17]. In case no consensus can be reached during the meeting, answer options can be modified or made less detailed to explore whether consensus could be reached on a more general level. In addition, one may choose to repeat the topic (with adjustments made) in a next meeting. Ultimately, one may choose to retain a certain point from the current SOP if no consensus can be reached.

### 2.2 Live audience engagement meeting

An ideal method to gather audience responses quickly and to subsequently discuss the answers within one meeting, is the use of live audience engagement platforms. Using this platform type, each expert can give her/his answer to a question, for example using their smartphone. After the answers are given, the (anonymous) results can be shown directly, for example in graphical form. Written commentary can be added to the answers anonymously to aid the discussion. The following discussion can be recorded, and experts are able to explain and motivate their answer. After discussion, the same question is repeated until consensus is reached. (Fig. 1, modified Digital Delphi round 2) Questions can be answered and discussed one-by-one, or, if questions have a more consecutive order, one may choose to first answer all (or a subgroup of) questions before revealing the results. In addition, questionnaires and responses can be exported to datasheets for further analysis if needed.

## Phase 3: presentation and implementation of the consensus-based protocol

### 3.1 Creating an SOP

After the live audience engagement meeting, results obtained from the questionnaire and discussions can be analyzed and agreements incorporated into a new SOP. The draft SOP is sent to the same group of experts and discussed in an online meeting to ensure expert group’s agreement. If agreement still exist, the surgeons pledge to adhere to the (updated) SOP, after which the SOP can be used for clinical routine. (Fig. 1, modified Digital Delphi round 3).

### 3.2 Evaluation of the new SOP

Evaluating the newly implemented SOP is important (to assess effectiveness) and can be done at several levels. For example, patient data should be recorded prospectively to evaluate whether the applied changes result in less complications. In addition, stakeholders can be interviewed to assess whether the newly implemented SOP is executable. At the next periodic SOP revision, these data should be analyzed by the moderator in the preparatory phase.

## Discussion

In this update, we explain how to reach consensus within a group of experts on healthcare-related problems where minimal evidence exists, using live audience engagement platforms. With this modified Digital Delphi method, one is able to create and implement SOP in clinical practice within a short period of time.

Standardization of surgical procedures is an important tool to increase efficacy and safety and has gained increased interest in recent years [18–20]. For example, implementation of SOP for cerebrospinal fluid shunt surgery reduced the rate of shunt infections significantly [8, 10]. The importance of creating and implementing SOPs has been explained in detail by Buis et al. [9]. They conclude that SOPs may lead to improved outcomes after surgery, at the price of some loss of individuality. Although we still agree with this statement, we think that this modified Digital Delphi technique retains the feeling of ownership, since the specialist’s opinion forms an integral part in the process, probably increasing protocol compliance. Moreover, feeling of ownership improves performance and satisfaction [21, 22].

There are some points of discussion on the methods we have applied: first, how does one define consensus? In our approach, we choose ≥ 80%, but this is arbitrary [16, 17, 23, 24]. Ideally, one may perform a sensitivity analysis investigating the influence of the consensus threshold on medical decisions. Second, to increase the compliance and usefulness of the newly formed SOP, we think it is important *not* to make too many changes at once, and keep the text short and simple, as addressed by Buis et al. [9], and seen in other aspects of quality improvement in healthcare [25]. This may be challenging, since we think it is also important to form a complete instruction guide to make it interpretable for (less experienced) trainees as well. To solve this problem, one can choose to divide the SOP into a few short and straightforward agreements upon which consensus was reached, and add in a separate section a more detailed (perioperative) guideline. Third, we modified the original Delphi technique since we think this a fast and pragmatic method to reach consensus, supported by readily available software. However, there are more described modifications to the original method, such as the ‘real-time Delphi’ or ‘policy Delphi’ [16, 26].

This method has not been described, studied or validated yet and some limitations should be addressed since discussions on the answers to the questionnaires are held directly, discussions could be dominated by verbally strong people or people higher in professional rank. This could influence other stakeholders to a greater extent compared to the original Delphi. This drawback is diminished by first writing arguments anonymously in the online environment, or repeating questions before discussion. Another drawback is that all stakeholders should be online at the same moment for the online discussions. Fourth, it is of great importance to register whether the implemented agreements are executable and lead to the desired outcomes, since only then the usefulness can be determined, and future improvements be made.

In the forthcoming years, with this method, we hope to contribute toward effective and safe consensus-based healthcare, despite lack of high-level evidence.

## Conclusion

Standardized, evidence-based care results in less complications. However, it can be challenging to reach consensus on topics where no or minimal evidences exist. In this update, we describe a method how to reach consensus within a group of experts through live audience engagement platforms, consensus can be reached in a short time, while the anonymous and interactive characteristics of the Delphi method are largely maintained.

Box: Real-life example of a consensus process regarding duraplasty during decompressive craniectomy **Phase 1**

**1.1 State scope of SOP, appointment of moderator**

The percentages of cerebrospinal fluid leakage, infections and epidural bleeding after cranioplasty are (too) high, which might be influenced by the decision whether to close the dura during the initial decompressive craniectomy or not. Therefore, as part of the cranioplasty and decompressive craniectomy SOPs, we aimed to reach consensus what surgical method to use during decompressive craniectomy considering dura closure. For this process, the first author (TG) was appointed as moderator.

**1.2 Problem identification: determine current surgical methods, evaluate literature**

We assessed surgical methods among our surgeons and residents and found different opinions and methods what to do with the opened dura during decompressive craniectomy. One option is to cover the cortical surface with the unapproximated dural flaps and absorbable hemostatic cellulose. Another option is to suture artificial dura or periosteum between the unapproximated flaps, thereby closing the dura without increasing intracranial pressure. The latter could be of importance to lower the chance on post-operative complications such as cerebrospinal fluid leakage. Furthermore, this possibly increases the successful use of tack-up sutures during subsequent cranioplasty at a later time, which is used to minimize epidural space. On the other hand, one may want to minimize surgery time in critically ill patients and extensive dura closure is time-consuming. Since there is no high-level evidence to support either of these approaches, a debate between neurosurgical experts could help to choose one approach.

**1.3 Selection of group of experts**

A group of experts was selected based on the following criteria: neurosurgeons and residents in training from our university hospital. The latter were considered as an expert too, since both decompressive craniectomy and cranioplasty are procedures performed often by neurosurgical residents, supervised by a neurosurgeon.

**1.4 Develop questionnaire (example of one of the questions)**

As part of the standardization process of both decompressive craniectomy and cranioplasty, one of the questions sent to the experts was whether the (enlarged) dura should (if possible) be closed during decompressive craniectomy. (Fig. 1, modified Digital Delphi round 1) Before the live audience engagement meeting, no further adjustments were made for this question.

**Phase 2**

**2.1 Defining consensus**

Before start of the interactive session, we set an agreement percentage of ≥ 80% per question as consensus. Questions that would reach this level of consensus were set to be implemented within the SOP. In case no consensus was reached, a modified question would be introduced in a second meeting. If no consensus was reached at the second meeting, the question would be excluded from the SOP at this moment.

**2.2 Live audience engagement meeting**

**Fig. 2:**
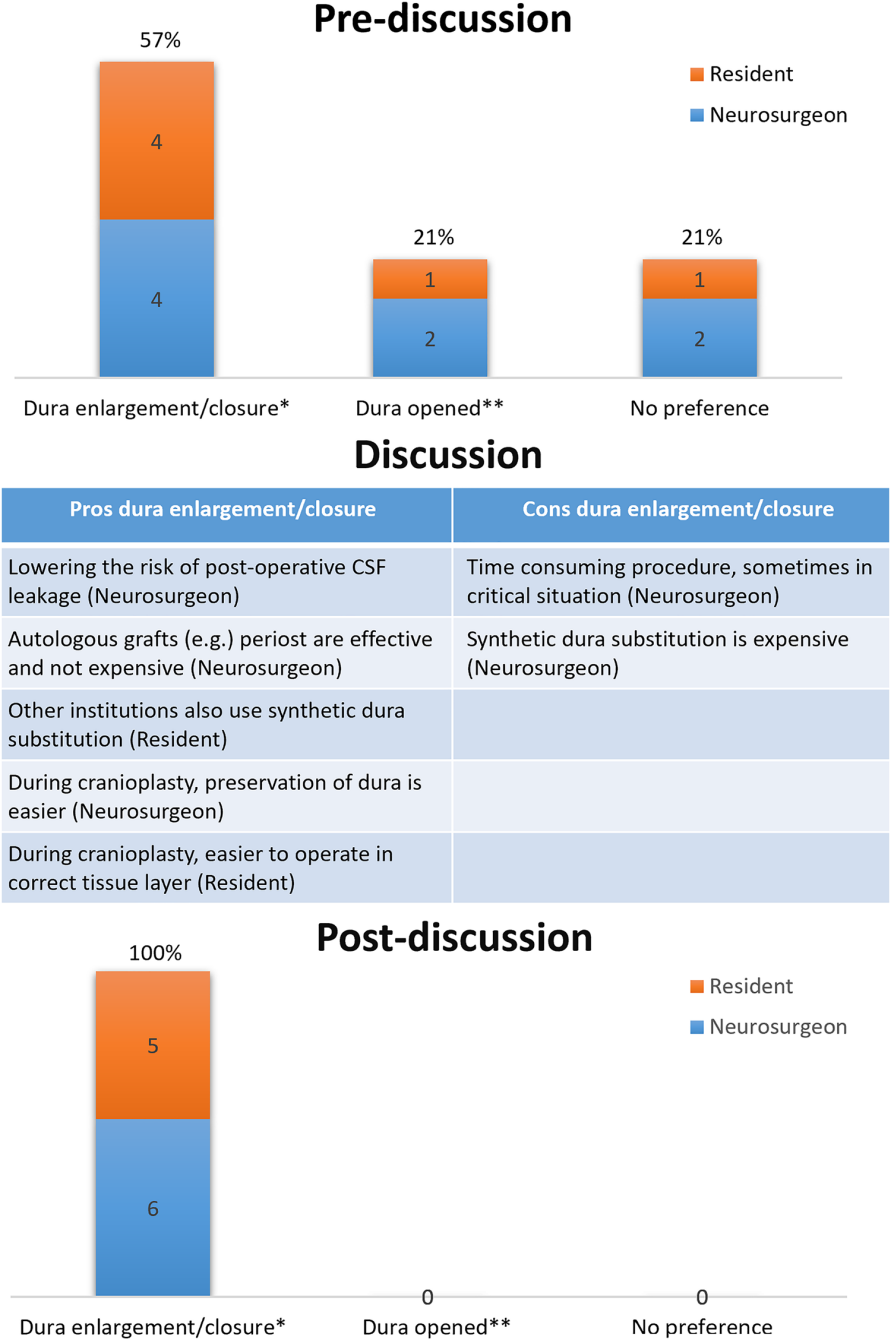
Example of one of the questions and outputs during the live audience engagement meeting: What to do with the opened dura during decompressive craniectomy? *Closure of the enlarged dura with either autologous grafts or synthetic dura substitution. **Leave the dura open, covered with absorbable hemostatic cellulose

As an interactive session, we chose to use AhaSlides (AhaSlides PTE. LTD., Singapore) as a live audience engagement platform. Each panel member registered and (individually) used a computer or smartphone to participate. In total, 14 (52% of invited) experts joined the meeting, of which eight neurosurgeons and six residents. Before discussion, 8/14 (57%) were in favor of dura closure, 3/14 (21%) had no preference, and 3/14 (21%) were in favor of leaving the dura open. After discussion, in which the possible merit of dura closure was discussed, the proportions changed to 11/11 (100%) voting for dura closure. (Fig. 2; NB All three experts who had no preference before discussion failed to respond to the question a second time for unknown reasons).

**Phase 3**

**3.1 Creating an SOP**

After analysis, the abovementioned results and implications for clinical practice were presented in a following meeting. (Fig. 1, modified Digital Delphi round 3) Since no further suggestions were made by the group of experts, we hereafter implemented the agreement to close the enlarged dura during decompressive craniectomy in our newly formed SOP.

**3.2 Evaluation**

In future, prospective patient data collection will reveal whether this has led to less complications during both decompressive craniectomy and cranioplasty.
